# Regulation of Microenvironments of Hydrogen-Bonded Organic Frameworks for Enhanced Enzyme Activity of Phosphotriesterase

**DOI:** 10.3390/molecules31101651

**Published:** 2026-05-14

**Authors:** Feier Wu, Peiyan Li, Yixuan Guo, Changsheng Du, Peng Li

**Affiliations:** 1 State Key Laboratory of Porous Materials for Separation and Conversion, Shanghai Key Laboratory of Molecular Catalysis and Innovative Materials, Department of Chemistry, College of Smart Materials and Future Energy, Fudan University, 2005 Songhu Road, Shanghai 200438, China; feierwu@fudan.edu.cn (F.W.); 23210220012@m.fudan.edu.cn (Y.G.); 2 Guangxi Key Laboratory of Clean Pulp & Papermakingand Pollution Control, School of Light Industry and Food Engineering, Guangxi University, Nanning 530004, China; 2416391022@st.gxu.edu.cn; 3 State Key Laboratory of Advanced Papermaking and Paper-Based Materials, School of Light Industry and Engineering, South China University of Technology, Guangzhou 510640, China

**Keywords:** hydrogen-bonded organic frameworks, phosphotriesterase, enzyme immobilization, microenvironment regulation, catalytic efficiency

## Abstract

The microenvironment of the porous channels in enzyme immobilization carriers critically determines the catalytic performance of immobilized enzymes. In this study, we systematically tuned the hydrophobicity/hydrophilicity of the channel microenvironment of hydrogen-bonded organic frameworks (HOFs) by introducing four different substituents (-CH_3_, -Cl, -F, -NH_2_) at the 2-position of the phenyl ring of the HOF-101 monomer. These HOF-101 derivatives, which are isostructural to the parent HOF-101, were used to immobilize phosphotriesterase (PTE). The enzyme loading efficiencies ranged from 64.7% to 70.7%, indicating that the substituents had little effect on PTE binding, which primarily relies on carboxyl-residue interactions. Kinetic studies revealed that the hydrophilic -NH_2_-functionalized HOF-101 (PTE@HOF-101-NH_2_) exhibited the highest catalytic efficiency (1.43 × 10^8^ M^−1^ s^−1^), 2.27 times that of free PTE, while the hydrophobic -CH_3_ analogue showed reduced activity. Notably, PTE@HOF-101-F demonstrated superior acid resistance (70% relative activity at pH 2) and long-term thermal stability (70% activity retention after 6 h at 70 °C), outperforming other derivatives. In contrast, PTE@HOF-101-NH_2_ showed the highest activity under mild conditions but suffered from framework dissolution under prolonged harsh treatments. This work demonstrates that fine-tuning the HOF channel microenvironment is an effective strategy to enhance enzyme activity and stability, providing a platform for designing advanced immobilized enzyme systems.

## 1. Introduction

Enzymes, as nature’s biocatalysts, exhibit unparalleled catalytic efficiency and substrate specificity under mild conditions. However, their practical applications are often hampered by fragile conformations, poor long-term operational stability, and low tolerance to harsh environments such as extreme pH, high temperature, and organic solvents [1,2,3]. Enzyme immobilization within solid porous matrices has emerged as a powerful strategy to address these challenges, offering enhanced stability, easy separation, and reusability [4,5].

Among various support materials, crystalline porous frameworks, including metal-organic frameworks (MOFs) and covalent organic frameworks (COFs), have attracted considerable attention due to their high surface areas, tunable pore sizes, and designable pore chemistries [6,7,8,9]. For instance, Liang and co-workers showed that hydrophilic MOF environments better preserve enzyme activity compared to hydrophobic ones [10]. Furthermore, multivariate MOF (MTV-MOF) strategies have been employed to continuously tune the pore hydrophilicity, revealing a non-linear relationship between framework hydrophilicity and encapsulated lipase activity, with an optimal transition point existing [11]. Similarly, pore wall hydrophobization in MOFs has been shown to induce the “open-lid” conformation of lipases, thereby boosting catalytic activity [12]. COFs, with their robust covalent linkages and ordered channels, have also been used to create confined microenvironments that enhance enzyme stability and substrate accessibility [13,14].

Despite these advances, several intrinsic limitations remain. Most MOF and COF syntheses require harsh conditions (e.g., high temperatures, organic solvents, or strong acids/bases), which can denature enzymes prior to or during immobilization [15,16]. Post-synthetic infiltration strategies, while milder, often suffer from low enzyme loading and incomplete pore occupancy due to diffusion limitations [17]. Moreover, the rigid and often hydrophobic nature of many MOF/COF pores can induce unfavorable conformational changes in enzymes, leading to activity loss [18,19]. The trade-off between stability and activity, where enhanced protection frequently comes at the cost of reduced catalytic efficiency, remains a central challenge in the field.

In this context, hydrogen-bonded organic frameworks (HOFs) have recently emerged as a promising alternative for enzyme immobilization [20,21]. HOFs are assembled from organic building blocks via non-covalent interactions (mainly hydrogen bonding and π-π stacking), offering several unique advantages: (i) exceptionally mild and aqueous synthesis conditions, which are highly biocompatible; (ii) structural diversity and easy functionalization without altering framework topology; and (iii) reversible assembly, allowing for defect healing and responsive behavior [22]. These features make HOFs particularly attractive for in situ encapsulation of enzymes under physiologically relevant conditions.

In our previous work, we reported the first in situ encapsulation of phosphotriesterase (PTE), an organophosphorus hydrolase, within a mesoporous HOF-101 framework [23]. By simply mixing the HOF-101 monomer (H_4_TABPy) with PTE in aqueous buffer, the framework self-assembled around the enzyme, yielding PTE@HOF-101 composites with ~70% loading efficiency. Remarkably, the immobilized PTE exhibited 1.60-fold higher catalytic activity than free PTE, along with significantly enhanced acid resistance, thermal stability, and reusability. This work demonstrated that HOFs can simultaneously address the activity-stability dilemma, a feat rarely achieved by MOFs or COFs.

Encouraged by these findings, we sought to further investigate how the pore microenvironment of HOFs influences the catalytic behavior of encapsulated enzymes. Given that the HOF-101 monomer bears a central pyrene core with four peripheral benzoic acid groups, the 2-position of the phenyl ring can be substituted with different functional groups without changing the overall framework topology. This provides an ideal platform to systematically modulate the hydrophilicity/hydrophobicity and functionalization of the pore interior. Here, we synthesized four isostructural HOF-101 derivatives bearing -CH_3_ (hydrophobic), -Cl (hydrophobic, moderately electronegative), -F (hydrophobic, strongly electronegative), and -NH_2_ (strongly hydrophilic) groups. Using these materials, we encapsulated PTE via the same mild in situ approach and systematically evaluated the resulting catalytic performance.

Compared to other enzyme immobilization platforms, HOFs offer several distinct advantages that motivated their selection in this study. Polyelectrolyte systems and silica gels, while widely used and cost-effective, typically lack the uniform, tunable pore architectures and crystalline order that enable systematic structure-activity relationship studies. The irregular pore size distribution and ill-defined surface chemistry of amorphous carriers make it difficult to decouple microenvironmental effects from mass transfer limitations. Metal-organic frameworks (MOFs) and covalent organic frameworks (COFs) provide well-defined pores but often require harsh synthesis conditions (e.g., organic solvents, high temperatures, strong acids/bases) that can denature enzymes prior to or during immobilization. In contrast, HOFs self-assemble under mild, aqueous, and biocompatible conditions, allowing direct in situ encapsulation of enzymes without activity loss. Furthermore, HOFs exhibit reversible assembly, which enables defect healing and responsive behavior—features not readily available in MOFs or COFs. Importantly, the isostructural nature of HOF-101 derivatives with different substituents at the 2-position provides an ideal platform for systematically varying pore hydrophobicity/hydrophilicity while keeping pore size, shape, and topology constant. This level of control is difficult to achieve with polyelectrolyte or silica-based systems, which often show batch-to-batch variability in pore structure. Thus, HOFs uniquely combine biocompatibility, structural precision, and chemical tunability, making them particularly suitable for mechanistic studies of microenvironment-enzyme activity relationships.

Here, we synthesized four isostructural HOF-101 derivatives bearing -CH_3_, -Cl, -F, and -NH_2_ groups, encapsulated PTE via mild in situ assembly, and systematically evaluated the resulting catalytic performance. This work aims to elucidate how pore hydrophobicity/hydrophilicity influences enzyme activity, stability, and recyclability.

## 2. Results and Discussion

### 2.1. Characterization of PTE@HOF-101 Derivatives

**Figure 1:**
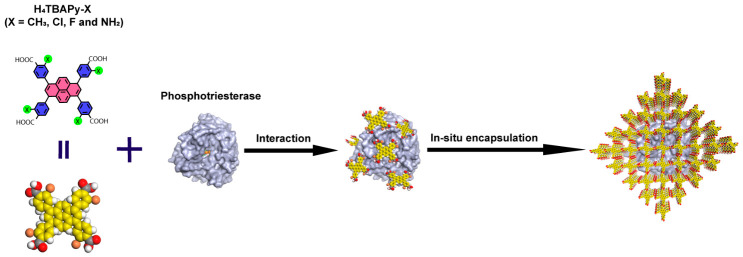
Synthetic process of PTE@HOF-101 derivatives.

**Figure 2:**
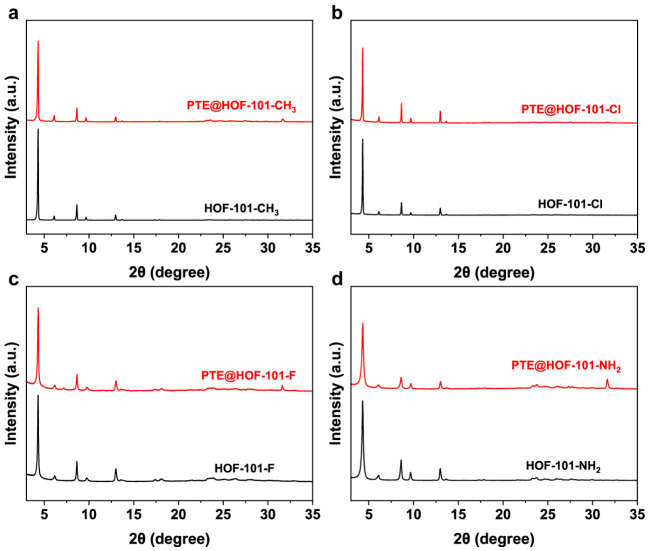
PXRD patterns of (**a**) HOF-101-CH_3_ and PTE@HOF-101-CH_2_; (**b**) HOF-101-Cl and PTE@HOF-101-Cl; (**c**) HOF-101-F and PTE@HOF-101-F; and (**d**) HOF-101-NH_2_ and PTE@HOF-101-NH_2_. Intensity is shown in arbitrary units (a.u.) for direct comparison of peak positions and relative crystallinity.

As shown in Figure 1, a series of PTE@HOF-101 derivatives (denoted as HOF-101-X, where X = CH_3_, Cl, F and NH_2_) were synthesized via a mild aqueous in situ self-assembly strategy. This approach facilitates the efficient encapsulation of PTE within the HOF matrices, circumventing the limitations typically imposed by the inherent pore size of the framework. The enzyme loading capacities for HOF-101-CH_3_, -Cl, -F and -NH_2_, were determined to be 64.7%, 68.5%, 66.2% and 70.7%, respectively (Figure S1). Powder X-ray diffraction (PXRD) patterns confirmed that all synthesized derivatives were isostructural to the parent HOF-101, with their crystalline integrity well-maintained following PTE incorporation (Figure S2 and Figure 2a–d). Furthermore, Fourier transform infrared (FT-IR) spectra exhibited characteristic amide I (1680–1630 cm^−1^) and amide II (1560–1530 cm^−1^) bands, verifying the presence of PTE (Figure S3). The successful encapsulation was further corroborated by Nitrogen sorption isotherms, which revealed a significant decrease in surface area, consistent with pore-blocking and mass shielding effects (Figure S4).

Scanning electron microscopy (SEM) confirmed that all PTE@HOF-101 derivatives retained the characteristic rod-like morphology (Figure S5). Confocal laser scanning microscopy (CLSM) was further employed to verify the spatial integration of PTE. The homogeneous red fluorescence arising from Rhodamine B-labeled PTE throughout the crystals was noted, illustrating deep enzyme penetration rather than superficial adsorption (Figure S6). This observation was further corroborated by STEM-HAADF imaging and energy-dispersive X-ray spectroscopy (EDS) mapping, which revealed a uniform distribution of N, S, and Zn atoms (characteristic elements of PTE) within the HOF matrix (Figures S7–S10).

### 2.2. Catalytic Performance

The catalytic performance of free PTE and PTE@HOF-101 derivatives was evaluated using the hydrolysis of dimethyl-(4-nitrophenyl) phosphate (DMNP) as a model reaction. The hydrolysis rate as a function of DMNP concentration was initially investigated (Figure S11). Control experiments confirmed that the pristine HOF-101-X exhibit negligible catalytic activity (Figure S12). The enzymatic kinetic parameters, including *K*_m_, *K*_max_, and *K*_cat_, were determined via the Michaelis-Menten model (Figure S13 and Table 1). PTE@HOF-101-NH_2_ exhibits the highest catalytic efficiency (*K*_cat_/*K*_m_ = 1.43 × 10^8^ M^−1^ s^−1^), 2.27 times that of free PTE. In contrast, the hydrophobic -CH_3_ derivative shows reduced efficiency (0.84-fold relative to free PTE). The order of catalytic efficiency was -NH_2_ > -F > -Cl > unmodified HOF-101 > free PTE > -CH_3_. This trend suggests a possible correlation between a hydrophilic channel microenvironment and enhanced catalytic efficiency. One plausible explanation is that improved wettability may aid in product desorption and facilitate water-mediated restoration of the PTE active site, although direct mechanistic evidence requires further investigation.

### 2.3. Stability Studies

**Figure 3:**
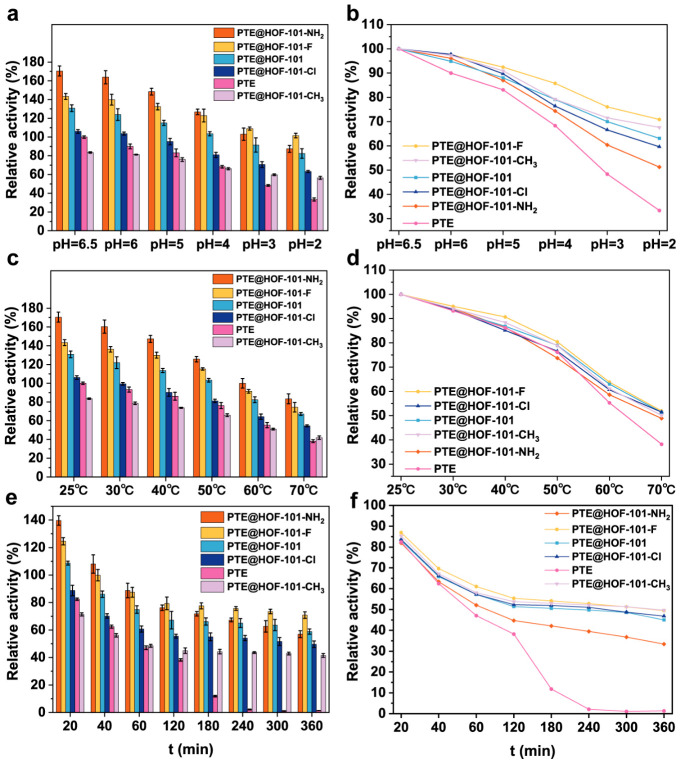
Relative activity of PTE@HOF-101 derivatives, free PTE, and HOF-101 (**a**,**b**) after 120 min incubation under different pH conditions; (**c**,**d**) after 120 min incubation at different temperatures; and (**e**,**f**) incubated at 70 °C for different time intervals. Note: In (**a**,**c**,**e**), all data are normalized to the activity of free PTE at 25 °C and pH 6.5. In (**b**,**d**,**f**), the initial activity of each specific sample (at 25 °C, pH 6.5) is set as 100% to reflect its relative stability.

To comprehensively evaluate how the tailored microenvironments influence the stability of PTE, we systematically investigated the stability of free and immobilized PTE under various conditions, including extreme pH, elevated temperatures, and prolonged incubation times. As depicted in Figure 3a, under acidic conditions (pH 2–6.5), all immobilized PTE derivatives retained higher activity than free PTE. Notably, PTE@HOF-101-F displayed high structural stability under acidic conditions, retaining approximately 70% of its initial activity at pH 2.0, whereas free PTE was almost completely inactivated (Figure 3b). Thermal stability assays at 70 °C further corroborated the advantage of the HOF-based encapsulation strategy (Figure 3c). At this temperature, all PTE@HOFs remained at approximately 50% of their initial activity, outperforming free PTE (Figure 3d). After incubation at 70 °C for 6 h, all PTE@HOF-101 derivatives exhibit significantly higher activity than free PTE (Figure 3e). Ultimately, most derivatives retained more than 50% of relative activity, whereas free PTE was almost completely inactivated. Notably, PTE@HOF-101-NH_2_ showed a more rapid activity decay than the other PTE@HOF-101 derivatives, possibly due to partial framework dissociation (Figure 3f).

**Figure 4:**
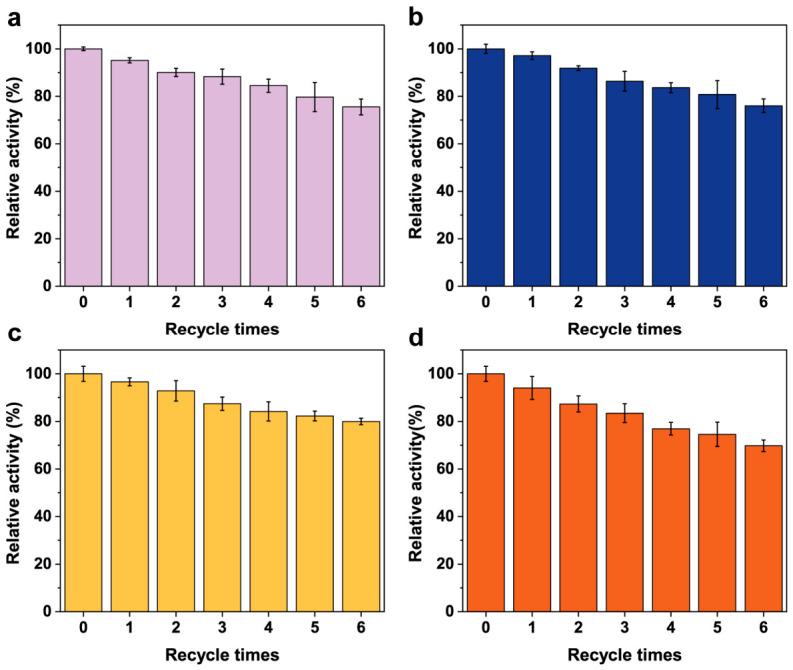
Recycling performance of (**a**) PTE@HOF-101-CH_3_; (**b**) PTE@HOF-101-Cl; (**c**) PTE@HOF-101-F; and (**d**) PTE@HOF-101-NH_2_.

Moreover, the recyclability of PTE@HOF-101 derivatives was investigated over six consecutive cycles (Figure 4a–d). PTE@HOF-CH_3_, PTE@HOF-Cl, and PTE@HOF-101-F all exhibit robust reusability, retaining at least 75% of their initial activity. Among them, PTE@HOF-101-F demonstrates the highest operational stability, preserving 80% of its catalytic capacity. In contrast, PTE@HOF-101-NH_2_ showed a more pronounced decrease in efficiency, dropping to 70% after the final cycle. To elucidate the mechanism of activity loss, we performed PXRD analysis on the recovered materials (Figure S14). The diffraction patterns of PTE@HOF-101-F remained sharp and well-defined, indicating high crystalline integrity. However, the PTE@HOF-101-NH_2_ samples exhibited noticeable peak broadening and intensity reduction. This structural evolution suggests that the high hydrophilicity of the -NH_2_-functionalized framework, while beneficial for kinetics, renders the lattice more susceptible to partial hydrolytic dissolution during repeated centrifugation and washing steps. These findings emphasize that the -F-modified microenvironment provides the optimal balance between catalytic promotion and structural longevity, making it a highly promising candidate for sustainable biocatalysis in demanding industrial environments.

## 3. Materials and Methods

### 3.1. Materials and Characterization

1,3,6,8-Tetrakis(4-carboxy-2-methylphenyl)pyrene (H_4_TABPy-CH_3_), 1,3,6,8-tetrakis(4-carboxy-2-chlorophenyl)pyrene (H_4_TABPy-Cl), 1,3,6,8-tetrakis(4-carboxy-2-fluorophenyl)pyrene (H_4_TABPy-F), and 1,3,6,8-tetrakis(4-carboxy-2-aminophenyl)pyrene (H_4_TABPy-NH_2_) were purchased from Extension Co., Ltd. (Changchun, China). Phosphate-buffered saline (PBS, pH 6.5) was obtained from Xibiao Co., Ltd. (Xiamen, China). Purified phosphotriesterase (PTE) was obtained via fermentation of the recombinant *E. coli* strain BL21/pTIG-trx-ob3. N,N-Dimethylformamide (DMF) and Rhodamine B were purchased from Sigma-Aldrich (Shanghai, China). Dimethyl-(4-nitrophenyl) phosphate (DMNP) was dissolved in acetone to prepare a 5 mg/mL stock solution.

The morphology of the crystals was characterized using a GeminiSEM 560 field-emission scanning electron microscope (SEM). Microstructures and elemental compositions were further analyzed using a Tecnai G2 F20 S-Twin field-emission transmission electron microscope (TEM) equipped with an energy-dispersive X-ray spectrometer (EDS). Powder X-ray diffraction (PXRD) patterns were recorded on a Smartlab 9kW diffractometer. Fourier transform infrared (FT-IR) spectra were collected using a Nicolet iS10 spectrometer. Nitrogen adsorption-desorption isotherms were measured at 77 K using a BSD-PS2 analyzer to determine the Brunauer-Emmett-Teller (BET) surface area and pore size distribution. Confocal laser scanning microscopy (CLSM) was employed to observe the spatial distribution of fluorescence. PTE concentrations were determined using the bicinchoninic acid (BCA) assay on a Cytation 3 multi-mode microplate reader.

### 3.2. Synthesis of HOF-101 Derivatives

For the synthesis of HOF-101-X (X = -CH_3_, -Cl, -F, -NH_2_), 110 mg of the purified monomer was dissolved in 11 mL of DMF and heated at 120 °C for 30 min to obtain a clear yellow solution. After cooling to room temperature, 10 mL of the solution was added to 90 mL of ethanol under stirring. The suspension was stirred for 12 h, and the precipitate was collected by centrifugation, washed three times with ethanol, and dried at room temperature.

### 3.3. Immobilization of PTE (PTE@HOF-101 Derivatives)

For each derivative, 60 mg of monomer was dissolved in 6 mL DMF at 120 °C for 30 min, filtered, and 5 mL of the solution was mixed rapidly with 5 mL of diluted PTE solution. The yellow precipitate formed immediately. After stirring at 150 rpm for 25 min and standing for 5 min, the mixture was centrifuged, washed with water and ethanol, and vacuum-dried. The product was dispersed in PBS buffer to match the free PTE concentration. Loading efficiencies were determined by BCA assay: 64.7% (CH_3_), 68.5% (Cl), 66.2% (F), and 70.7% (NH_2_).

### 3.4. Catalytic Activity Measurement

Catalytic activity was measured using DMNP as substrate in pH 6.5 PBS at 25 °C. Initial rates (V_0_) at various substrate concentrations were fitted to the Michaelis-Menten equation to obtain *K*_m_, *V*_max_, and *K*_cat_.

## 4. Conclusions

In summary, we successfully synthesized four isostructural HOF-101 derivatives with systematically varied channel hydrophobicity/hydrophilicity and used them to immobilize PTE. The hydrophilic -NH_2_-functionalized HOF-101 provided the highest catalytic efficiency (2.27-fold that of free PTE) under mild conditions, making it suitable for rapid detoxification. Beyond the immediate findings, this work provides several directions for future exploration. First, the isostructural HOF platform can be readily extended to immobilize other enzymes, such as organophosphorus hydrolase or lipases, to test whether the observed hydrophilicity-activity correlation is generalizable. Second, the introduction of additional functional groups (e.g., -OH, -COOH, -SO_3_H) may further expand the polarity spectrum and reveal optimal microenvironments for specific biocatalytic reactions. Third, the mild and aqueous assembly conditions of HOFs offer a unique opportunity to co-encapsulate multiple enzymes within a single framework, potentially enabling cascade reactions in confined nanospaces. From an application perspective, the exceptional acid and thermal stability of PTE@HOF-101-F positions it as a promising candidate for organophosphate detoxification in chemically harsh industrial or environmental settings.

## Figures and Tables

**Table 1:** Kinetic parameters of free PTE and PTE@HOF-101 derivatives.

Sample	*V*_max_ (μM s^−1^)	*K*_m_ (μM)	*K*_cat_ (s^−1^)	*K*_cat_/*K*_m_ (M^−1^ s^−1^)
PTE@HOF-101-NH_2_	1.29	0.039	5.58	1.43 × 10^8^
PTE@HOF-101-F	1.12	0.045	4.81	1.08 × 10^8^
PTE@HOF-101	1.08	0.053	4.64	8.79 × 10^7^
PTE@HOF-101-Cl	0.84	0.048	3.63	7.64 × 10^7^
Free PTE	0.82	0.056	3.54	6.30 × 10^7^
PTE@HOF-101-CH_3_	0.68	0.056	2.93	5.27 × 10^7^

## Data Availability

The original contributions presented in the study are included in the article. Further inquiries can be directed to the corresponding author.

## Supplementary Materials

The following supporting information can be downloaded at: https://www.mdpi.com/article/10.3390/molecules31101651/s1, **Figure S1:** Determination of the embedding rate of PTE in (**a**) HOF-101-CH_3_; (**b**) HOF-101-Cl; (**c**) HOF-101-F; (**d**) HOF-101-NH_2_; **Figure S2:** PXRD patterns of HOF-101 and its derivatives; **Figure S3:** FT-IR spectra of PTE and (**a**) PTE@HOF-101-CH_3_; (**b**) PTE@HOF-101-Cl; (**c**) PTE@HOF-101-F; (**d**) PTE@HOF-101-NH_2_; **Figure S4:** Nitrogen adsorption-desorption isotherms of HOF-101-X and PTE@HOF-101-X. (**a**) X = CH_3_; (**b**) X = Cl; (**c**) X = F; (**d**) X = NH_2_; **Figure S5:** SEM images of HOF-101-X and PTE-HOF-101-X. (**a**,**b**) X = CH_3_; (**c**,**d**) X = Cl; (**e**,**f**) X = F; (**g**,**h**) X = NH_2_; **Figure S6:** CLSM images of Rhodamine B-labeled PTE-HOF-101-X. (**a**) X = CH_3_; (**b**) X = Cl; (**c**) X = F; (**d**) X = NH_2_; **Figure S7:** STEM-HAADF image of PTE@HOF-101-CH_3_ and corresponding EDS elemental mapping of C, O, N, S, and Zn; **Figure S8:** STEM-HAADF image of PTE@HOF-101-Cland corresponding EDS elemental mapping of C, O, N, S, and Zn; **Figure S9:** STEM-HAADF image of PTE@HOF-101-Fand corresponding EDS elemental mapping of C, O, N, S, and Zn; **Figure S10:** STEM-HAADF image of PTE@HOF-101-NH_2_ and corresponding EDS elemental mapping of C, O, N, S, and Zn; **Figure S11:** The catalytic kinetics curves of (**a**) PTE@HOF-101-CH_3_; (**b**) PTE@HOF-101-Cl; (**c**) PTE@HOF-101-F and (**d**) PTE@HOF-101-NH_2_ under different concentration of DMNP; **Figure S12:** The catalytic kinetics curves of PTE@HOF-101-X, and HOF-101-Xunder 0.16 mM of DMNP. (**a**) X = CH_3_; (**b**) X = Cl; (**c**) X = F; (**d**) X = NH_2_; **Figure S13:** (**a**) Initial reaction rates (*V*_0_) of PTE@HOF-101 derivatives, PTE@HOF-101, and free PTE as a function of substrate DMNP concentration. The data were fitted using the Michaelis-Menten model to determine the catalytic kinetic parameters; (**b**) Comparison of the catalytic efficiencies of PTE@HOF-101 derivatives, PTE@HOF-101, and free PTE; **Figure S14:** PXRD patterns of PTE@HOF-101-Xbefore and after cycling. (**a**) X = CH_3_; (**b**) X = Cl; (**c**) X = F; (**d**) X = NH_2_. **Table S1.** Comparison of the key characteristics of the composites.

## References

[1] Klibanov A.M. (1983). Immobilized Enzymes and Cells as Practical Catalysts. Science.

[2] Liang W., Wied P., Carraro F., Sumby C.J., Nidetzky B., Tsung C.-K., Falcaro P., Doonan C.J. (2021). Metal–Organic Framework-Based Enzyme Biocomposites. Chem. Rev..

[3] Sheldon R.A., van Pelt S. (2013). Enzyme immobilisation in biocatalysis: Why, what and how. Chem. Soc. Rev..

[4] Lian X., Fang Y., Joseph E., Wang Q., Li J., Banerjee S., Lollar C., Wang X., Zhou H.-C. (2017). Enzyme–MOF (metal–organic framework) composites. Chem. Soc. Rev..

[5] Huang S., Chen G., Ouyang G. (2022). Confining enzymes in porous organic frameworks: From synthetic strategy and characterization to healthcare applications. Chem. Soc. Rev..

[6] Chen Y., Jiménez-Ángeles F., Qiao B., Krzyaniak M.D., Sha F., Kato S., Gong X., Buru C.T., Chen Z., Zhang X. (2020). Insights into the Enhanced Catalytic Activity of Cytochrome c When Encapsulated in a Metal–Organic Framework. J. Am. Chem. Soc..

[7] Li P., Moon S.-Y., Guelta M.A., Harvey S.P., Hupp J.T., Farha O.K. (2016). Encapsulation of a Nerve Agent Detoxifying Enzyme by a Mesoporous Zirconium Metal–Organic Framework Engenders Thermal and Long-Term Stability. J. Am. Chem. Soc..

[8] Sun Q., Fu C.-W., Aguila B., Perman J., Wang S., Huang H.-Y., Xiao F.-S., Ma S. (2018). Pore Environment Control and Enhanced Performance of Enzymes Infiltrated in Covalent Organic Frameworks. J. Am. Chem. Soc..

[9] Li P., Modica J.A., Howarth A.J., Vargas E., Moghadam P.Z., Snurr R.Q., Mrksich M., Hupp J.T., Farha O.K. (2016). Toward Design Rules for Enzyme Immobilization in Hierarchical Mesoporous Metal-Organic Frameworks. Chem.

[10] Liang W., Xu H., Carraro F., Maddigan N.K., Li Q., Bell S.G., Huang D.M., Tarzia A., Solomon M.B., Amenitsch H. (2019). Enhanced Activity of Enzymes Encapsulated in Hydrophilic Metal–Organic Frameworks. J. Am. Chem. Soc..

[11] Li Y.-M., Yuan J., Ren H., Ji C.-Y., Tao Y., Wu Y., Chou L.-Y., Zhang Y.-B., Cheng L. (2021). Fine-Tuning the Micro-Environment to Optimize the Catalytic Activity of Enzymes Immobilized in Multivariate Metal–Organic Frameworks. J. Am. Chem. Soc..

[12] Guo L., He R., Chen G., Yang H., Kou X., Huang W., Gao R., Huang S., Huang S., Zhu F. (2024). A Synergetic Pore Compartmentalization and Hydrophobization Strategy for Synchronously Boosting the Stability and Activity of Enzyme. J. Am. Chem. Soc..

[13] Li M., Qiao S., Zheng Y., Andaloussi Y.H., Li X., Zhang Z., Li A., Cheng P., Ma S., Chen Y. (2020). Fabricating Covalent Organic Framework Capsules with Commodious Microenvironment for Enzymes. J. Am. Chem. Soc..

[14] Xing C., Mu Z., Li B., Yang J., Feng X., Zhang Y., Wang B. (2025). Tailoring Artificial Hydration Microenvironments in Covalent Organic Frameworks for Enhanced Enzymatic Catalysis in Organic Media. J. Am. Chem. Soc..

[15] Liang K., Ricco R., Doherty C.M., Styles M.J., Bell S., Kirby N., Mudie S., Haylock D., Hill A.J., Doonan C.J. (2015). Biomimetic mineralization of metal-organic frameworks as protective coatings for biomacromolecules. Nat. Commun..

[16] Chen G., Huang S., Ma X., He R., Ouyang G. (2023). Encapsulating and stabilizing enzymes using hydrogen-bonded organic frameworks. Nat. Protoc..

[17] Doonan C., Riccò R., Liang K., Bradshaw D., Falcaro P. (2017). Metal–Organic Frameworks at the Biointerface: Synthetic Strategies and Applications. Acc. Chem. Res..

[18] Chen G., Kou X., Huang S., Tong L., Shen Y., Zhu W., Zhu F., Ouyang G. (2020). Modulating the Biofunctionality of Metal–Organic-Framework-Encapsulated Enzymes through Controllable Embedding Patterns. Angew. Chem. Int. Ed..

[19] Hu Y., Dai L., Liu D., Du W. (2018). Rationally designing hydrophobic UiO-66 support for the enhanced enzymatic performance of immobilized lipase. Green. Chem..

[20] Chen G., Huang S., Shen Y., Kou X., Ma X., Huang S., Tong Q., Ma K., Chen W., Wang P. (2021). Protein-directed, hydrogen-bonded biohybrid framework. Chem.

[21] Huang S., Li J., Lin Y., Tong L., Zhong N., Huang A., Ma X., Huang S., Yi W., Shen Y. (2024). Hydrogen-Bonded Supramolecular Nanotrap Enabling the Interfacial Activation of Hosted Enzymes. J. Am. Chem. Soc..

[22] Yu D., Zhang H., Ren J., Qu X. (2023). Hydrogen-bonded organic frameworks: New horizons in biomedical applications. Chem. Soc. Rev..

[23] Guo Y., Mo G., Deng Y., Bi Y., Li P. (2026). In Situ Encapsulation of Phosphotriesterases by a Mesoporous Hydrogen-Bonded Organic Framework Engenders Enhanced Activity and Stability. ACS Appl. Mater. Interfaces.

