# Supplementary material for: Regulation of Microenvironments of Hydrogen-Bonded Organic Frameworks for Enhanced Enzyme Activity of Phosphotriesterase
Source: Molecules. 2026 May 14;31(10):1651. doi: 10.3390/molecules31101651 (PMC13210137; doi:10.3390/molecules31101651)
Supplement: Supplementary file 1 [file molecules-31-01651-s001.zip › molecules-4296354-supplementary.pdf]

Supporting Information

Regulation of Microenvironments of Hydrogen-Bonded Organic Frameworks for Enhanced Enzyme Activity of Phosphotriesterase

Feier Wu [1], Peiyan Li [2], Yixuan Guo [1], Changsheng Du [3,*] and Peng Li [1,*]

[1] State Key Laboratory of Porous Materials for Separation and Conversion, Shanghai Key Laboratory of Molecular Catalysis and Innovative Materials, Department of Chemistry, College of Smart Materials and Future Energy, Fudan University, 2005 Songhu Road, Shanghai 200438, China; feierwu@fudan.edu.cn (F.W.); 23210220012@m.fudan.edu.cn (Y.G.)

[2] Guangxi Key Laboratory of Clean Pulp & Papermakingand Pollution Control, School of Light Industry and Food Engineering, Guangxi University, Nanning 530004, China; 2416391022@st.gxu.edu.cn

[3] State Key Laboratory of Advanced Papermaking and Paper-Based Materials, School of Light Industry and Engineering, South China University of Technology, Guangzhou 510640, China

* Correspondence: 20269087@scut.edu.cn (C.D.); penglichem@fudan.edu.cn (P.L.)

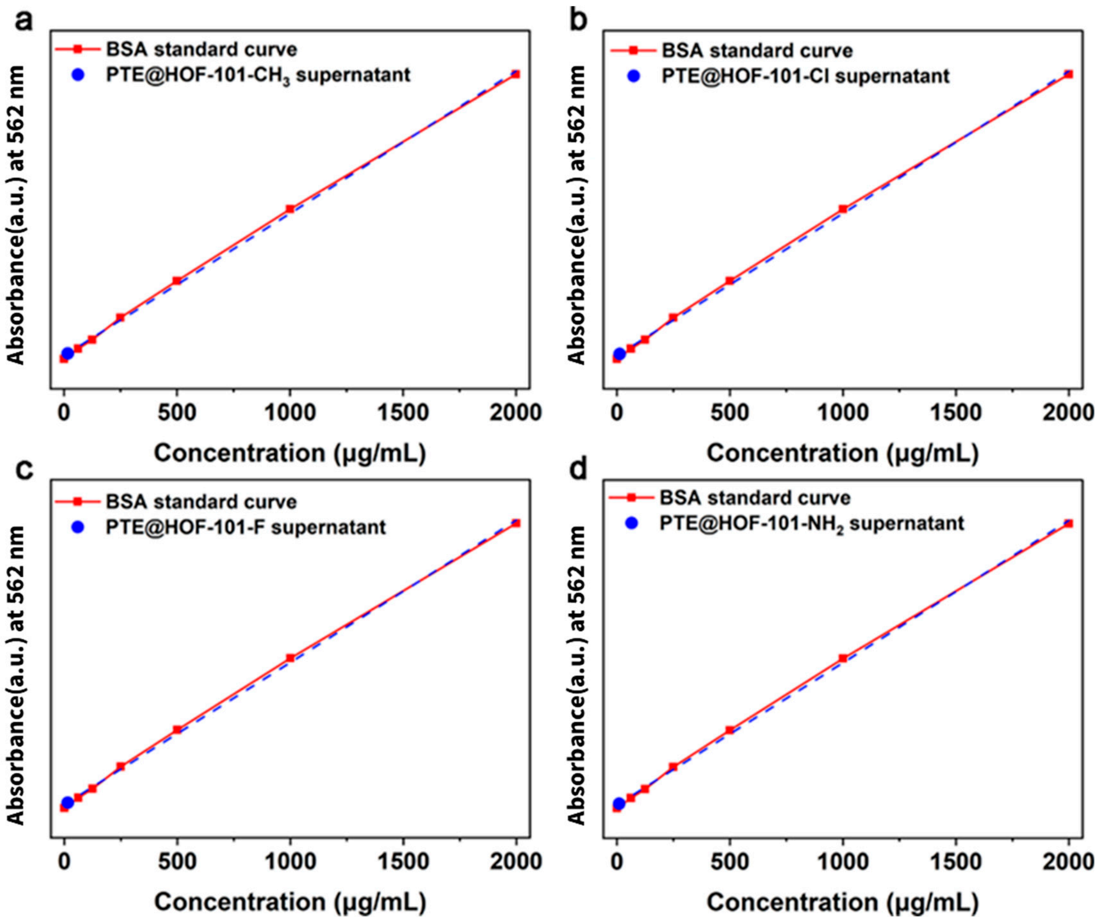

Figure S1. Determination of the embedding rate of PTE in (a) HOF-101-CH_3, (b) HOF-101-Cl, (c) HOF-101-F, (d) HOF-101-NH_2.

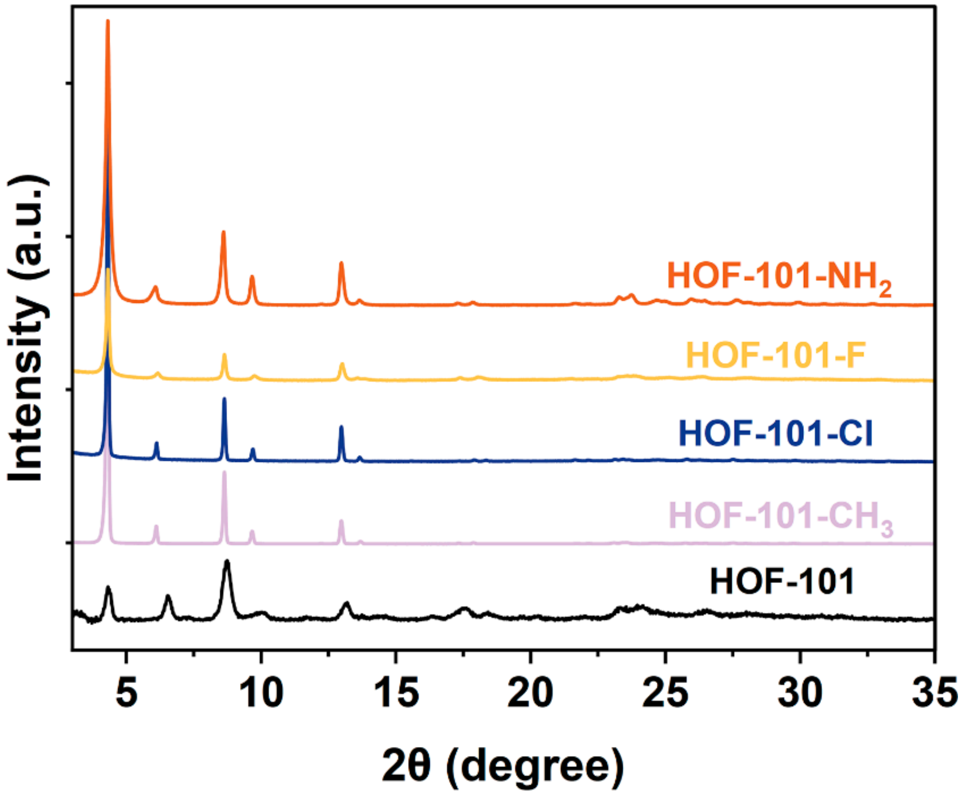

Figure S2. PXRD patterns of HOF-101 and its derivatives. Intensity is shown in arbitrary units (a.u.) for direct comparison of peak positions and relative crystallinity.

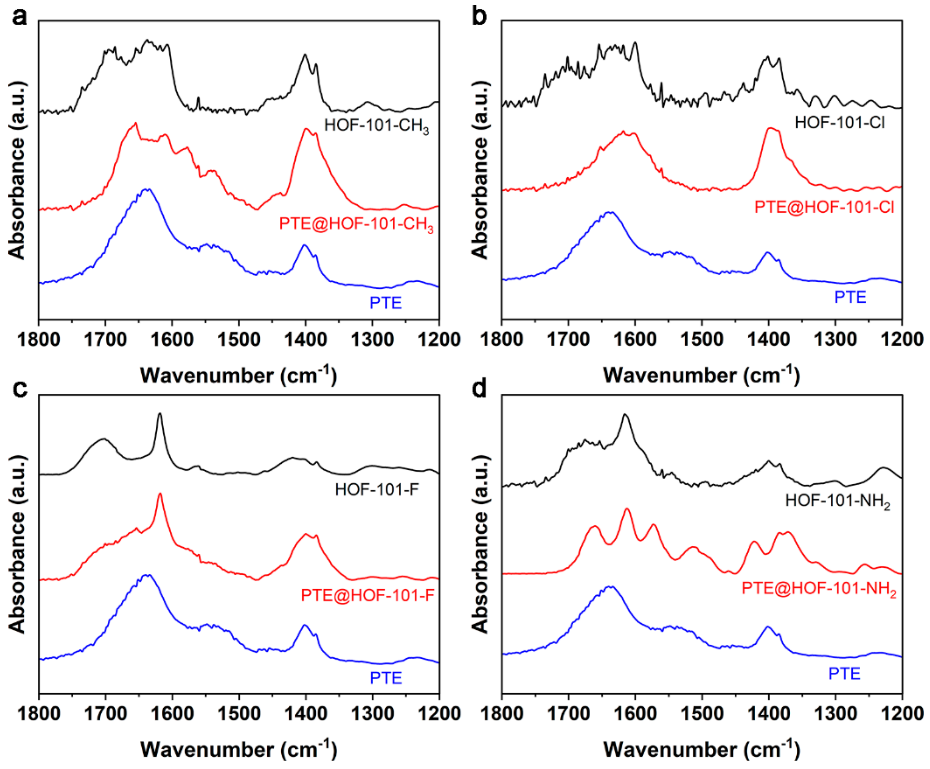

Figure S3. FT-IR spectra of PTE and (a) PTE@HOF-101-CH_3, (b) PTE@HOF-101-Cl, (c) PTE@HOF-101-F, (d) PTE@HOF-101-NH_2.

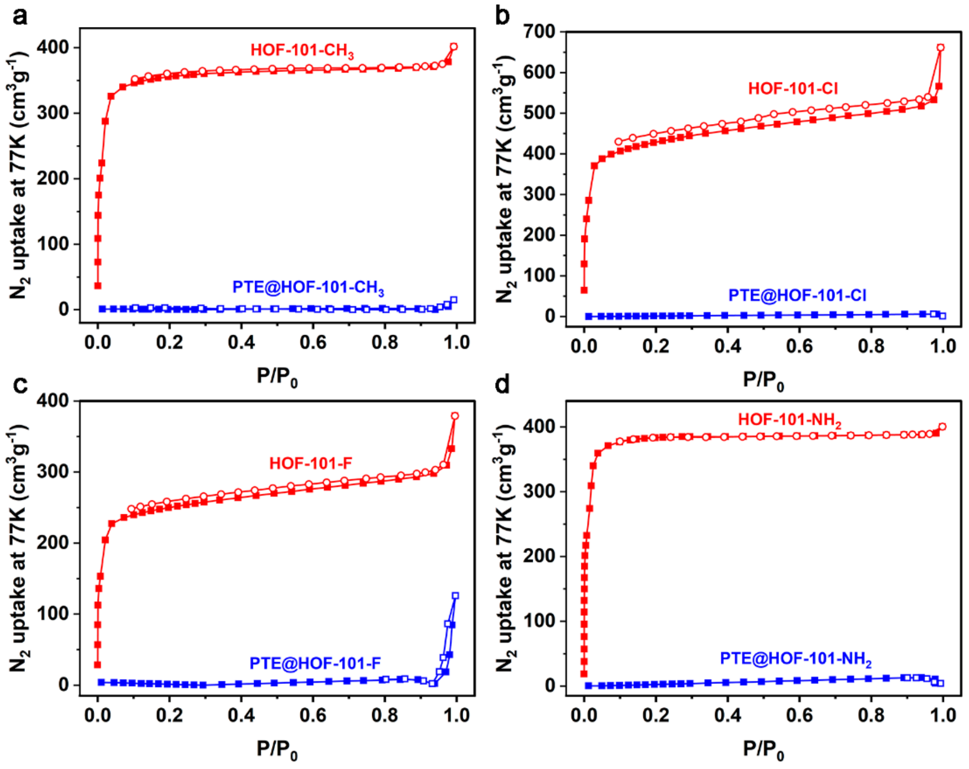

Figure S4. Nitrogen adsorption-desorption isotherms of HOF-101-X and PTE@HOF-101-X. (a) X = CH_3; (b) X = Cl; (c) X = F; (d) X = NH_2.

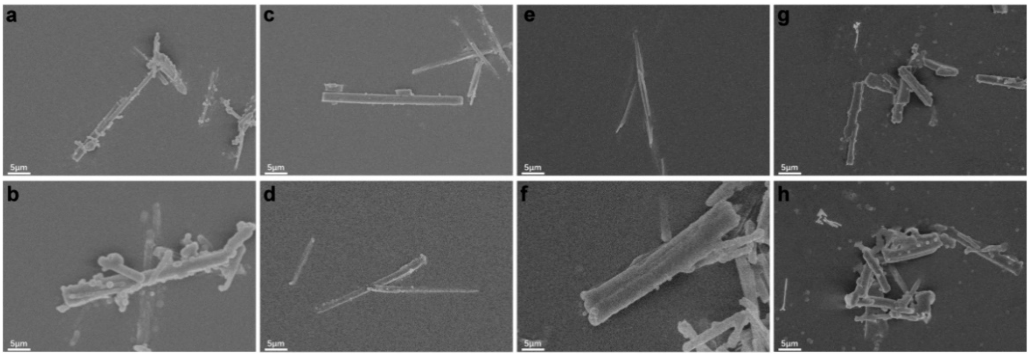

Figure S5. SEM images of HOF-101-X and PTE-HOF-101-X. (a, b) X = CH_3; (c, d) X = Cl; (e, f) X = F; (g, h) X = NH_2.

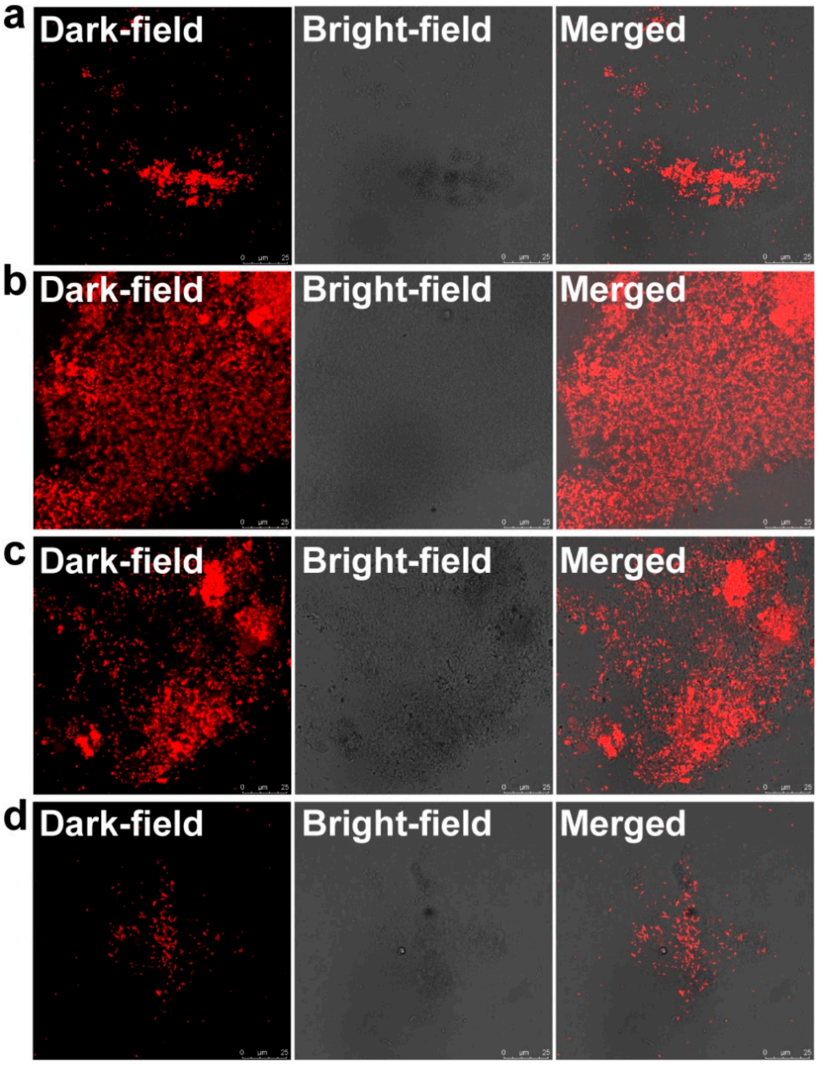

Figure S6. CLSM images of Rhodamine B-labeled PTE-HOF-101-X. (a) X = CH3; (b) X = Cl; (c) X = F; (d) X = NH2.

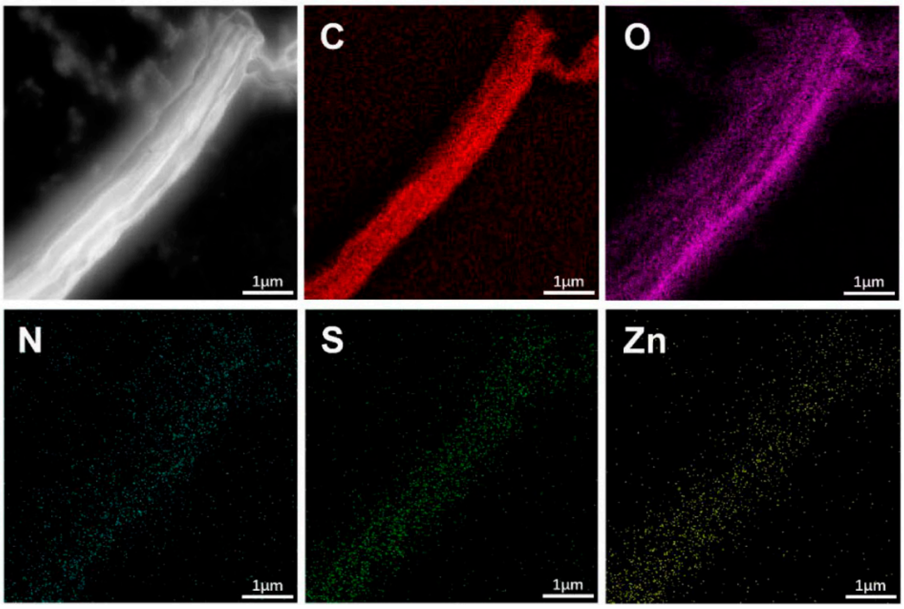

Figure S7. STEM-HAADF image of PTE@HOF-101-CH_3 and corresponding EDS elemental mapping of C, O, N, S, and Zn.

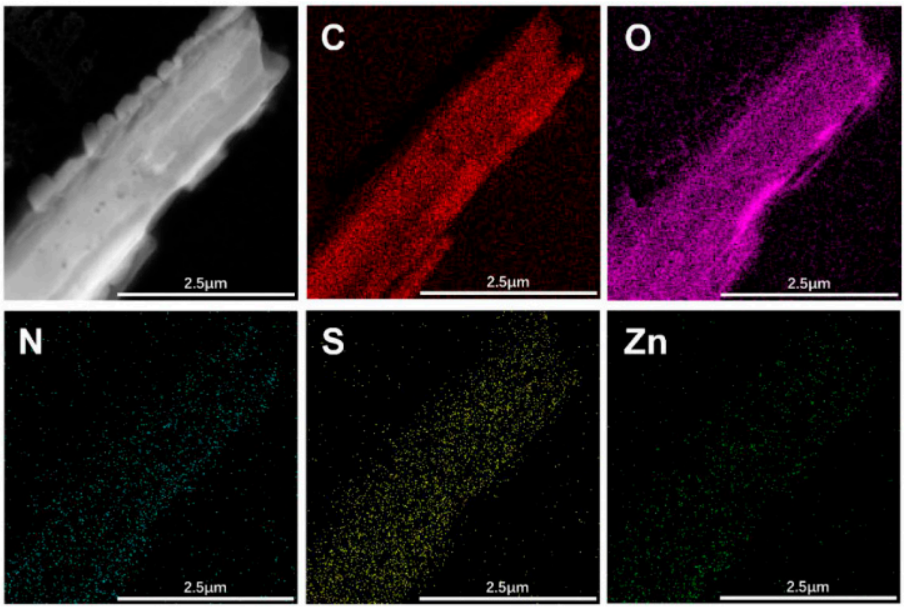

Figure S8. STEM-HAADF image of PTE@HOF-101-Cl and corresponding EDS elemental mapping of C, O, N, S, and Zn.

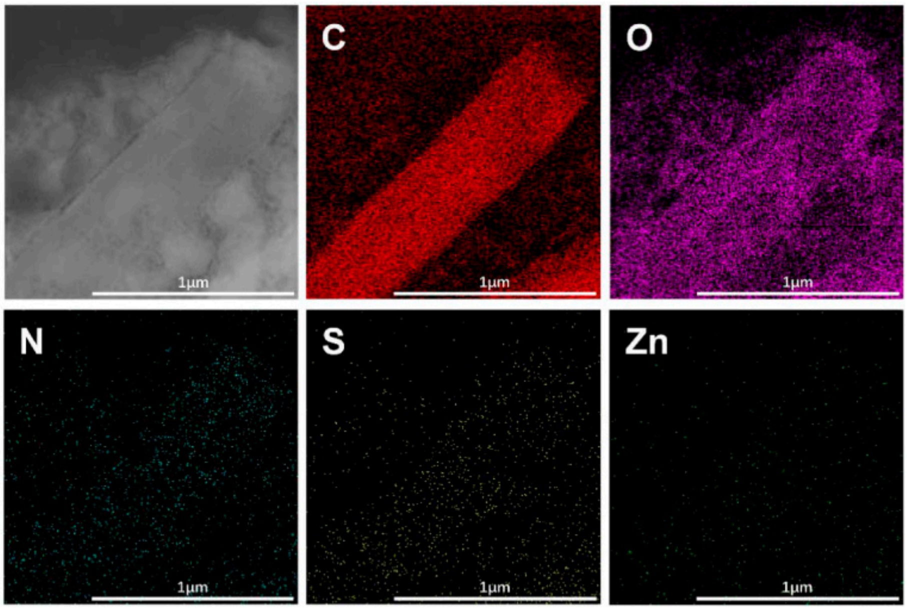

Figure S9. STEM-HAADF image of PTE@HOF-101-F and corresponding EDS elemental mapping of C, O, N, S, and Zn.

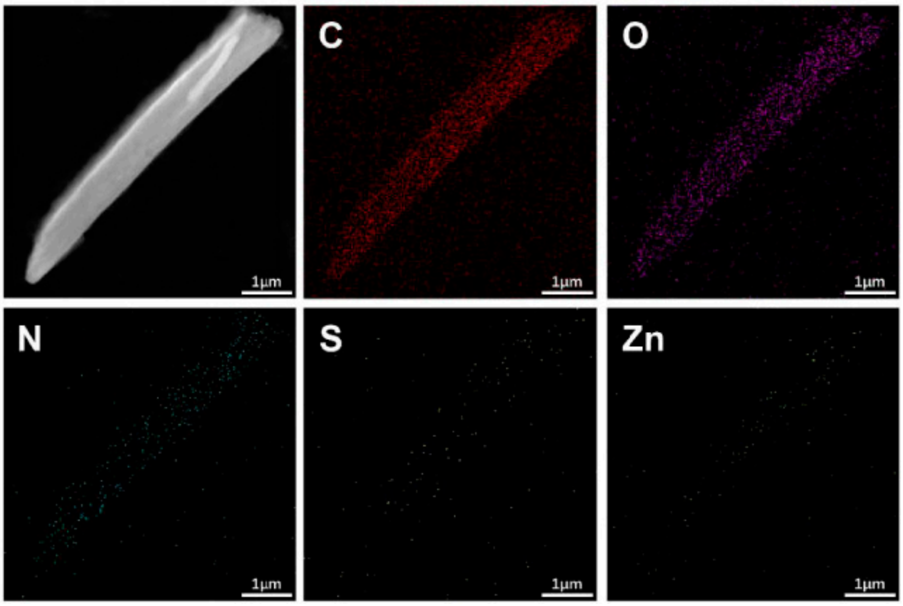

Figure S10. STEM-HAADF image of PTE@HOF-101-NH_2 and corresponding EDS elemental mapping of C, O, N, S, and Zn.

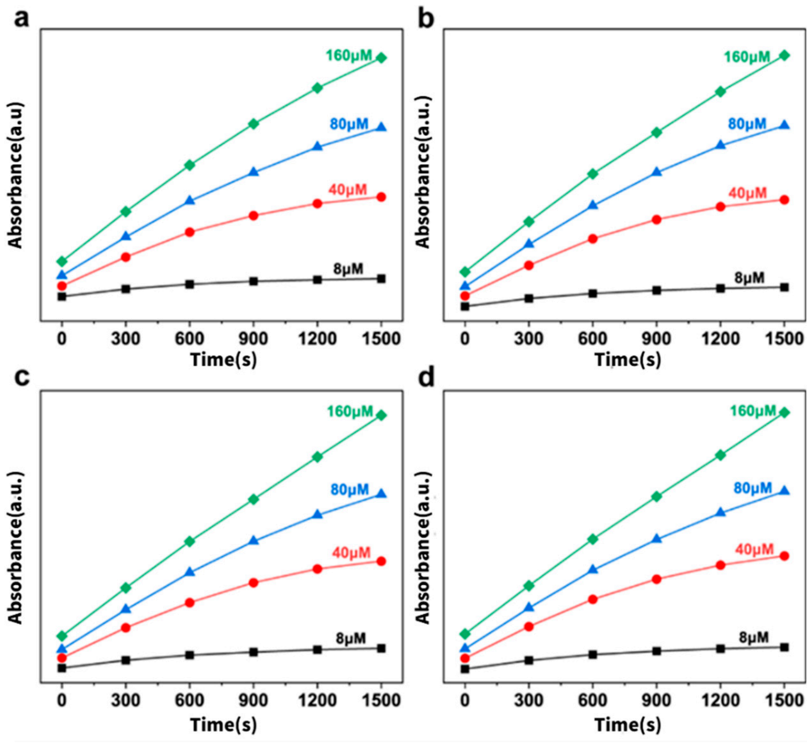

Figure S11. The catalytic kinetics curves of (a) PTE@HOF-101-CH_3; (b) PTE@HOF-101-Cl; (c) PTE@HOF-101-F and (d) PTE@HOF-101-NH_2 under different concentration of DMNP.

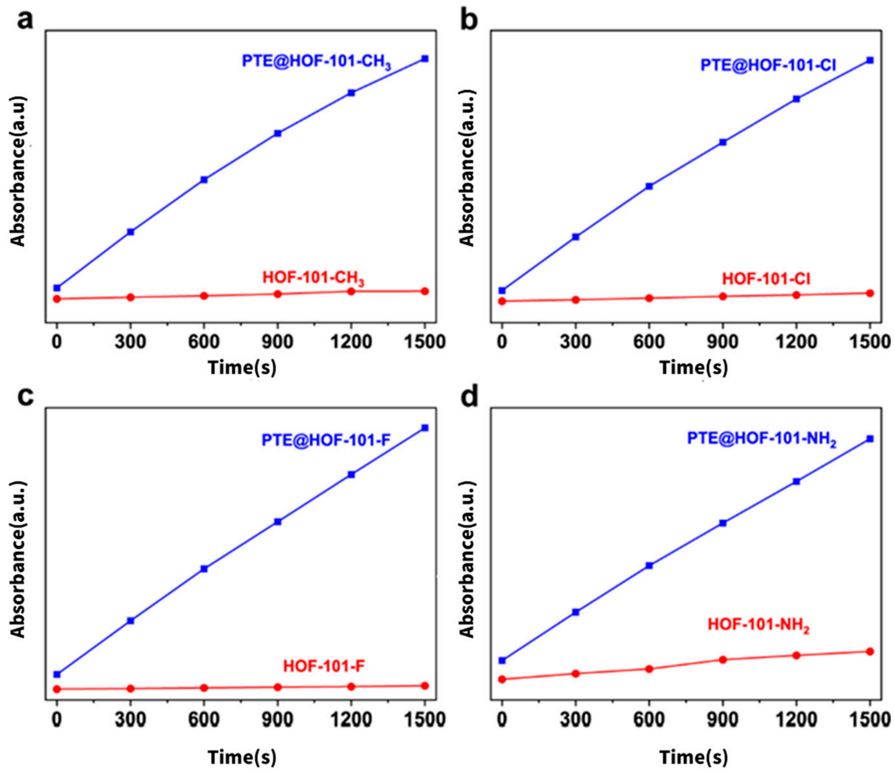

Figure S12. (a) Initial reaction rates (V_0) of PTE@HOF-101 derivatives, PTE@HOF-101, and free PTE as a function of substrate DMNP concentration. The data were fitted using the Michaelis–Menten model to determine the catalytic kinetic parameters. (b) Comparison of the catalytic efficiencies of PTE@HOF-101 derivatives, PTE@HOF-101, and free PTE.

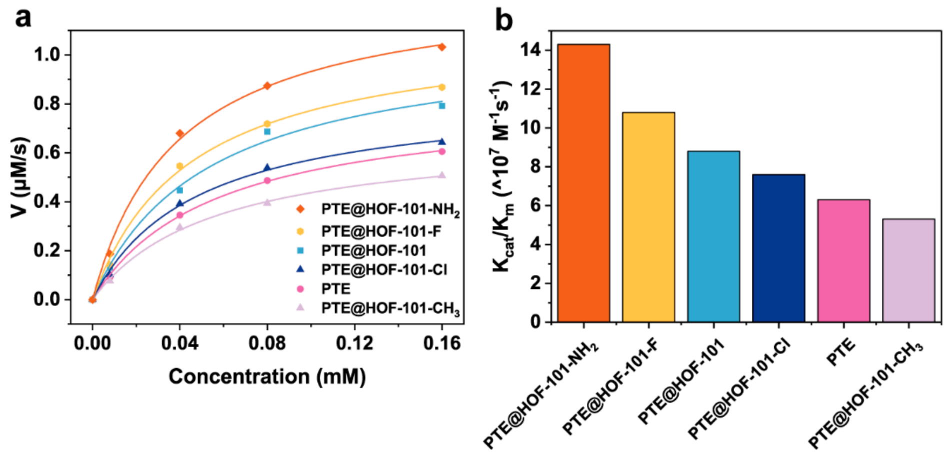

Figure S13. The catalytic kinetics curves of PTE@HOF-101-X, and HOF-101-X under 0.16mM of DMNP. (a) X = CH_3; (b) X = Cl; (c) X = F; (d) X = NH_2.

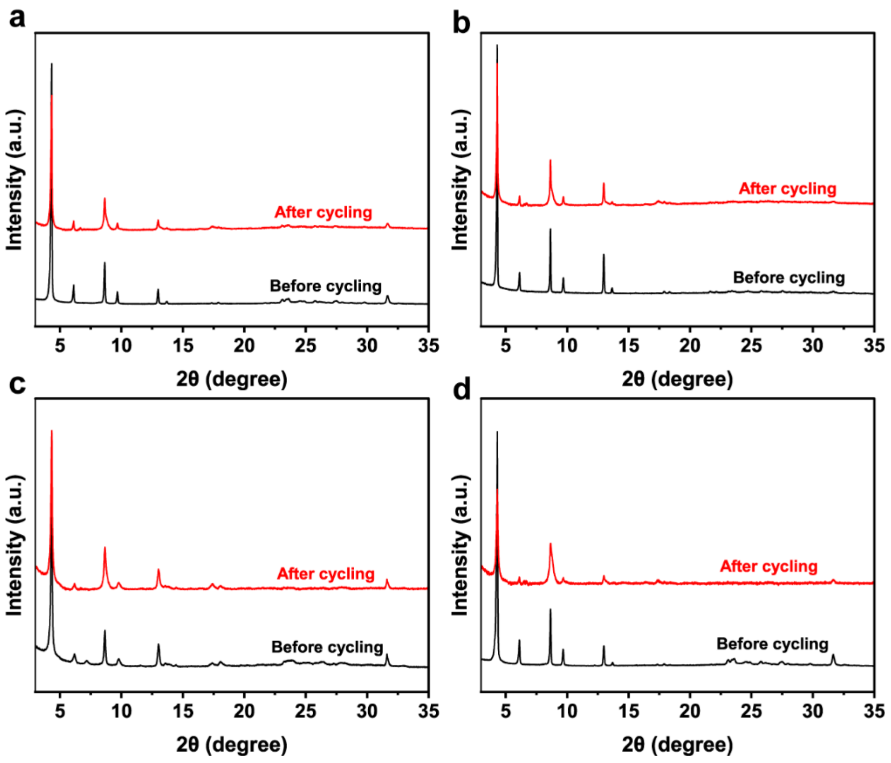

Figure S14. PXRD patterns of PTE@HOF-101-X before and after cycling. (a) X = CH_3; (b) X = Cl; (c) X = F; (d) X = NH_2. Intensity is shown in arbitrary units (a.u.) for direct comparison of peak positions and relative crystallinity.

Table S1. Comparison of the key characteristics of the composites

	PTE@HOF-101-CH_3	PTE@HOF-101-Cl	PTE@HOF-101-F	PTE@HOF-101-NH_3
Enzyme loading capacitye	64.7%	68.5%	66.2%	70.7%
